# Association of antibiotic exposure with the mortality in metastatic colorectal cancer patients treated with bevacizumab-containing chemotherapy: A hospital-based retrospective cohort study

**DOI:** 10.1371/journal.pone.0221964

**Published:** 2019-09-10

**Authors:** Linbin Lu, Tingting Zhuang, Erqian Shao, Yanhong Liu, Huimin He, Zhimin Shu, Yan Huang, Yichen Yao, Shan Lin, Shaoqin Lin, Xi Chen, Xiong Chen

**Affiliations:** 1 Department of Oncology, Fuzhou General Hospital of Nanjing Military Command, Fuzong Clinical College of Fujian Medical University, Fuzhou, Fujian, PR China; 2 Department of Neurology, Fuzhou General Hospital of Nanjing Military Command, Fuzhou, Fujian, PR China; Chang Gung Memorial Hospital at Linkou, TAIWAN

## Abstract

**Background:**

Preclinical studies showed that antibiotic exposure played a role in clinical outcomes in patients with chemotherapy via modulation of microbiota. However, it remains unknown whether antibiotic exposure during the bevacizumab therapy affects the clinical outcomes in metastatic colorectal cancer(mCRC) patients. This study aimed to examine the association between the antibiotic medication and the clinical outcomes in mCRC patients with bevacizumab therapy.

**Methods:**

This retrospective cohort analysis included 147 mCRC patients treated with bevacizumab. The hazard ratio of death was estimated using three Cox proportional hazards models with (1) never vs ever; (2) never vs 1–6 days and 7–40 days;(3) increase per day, and further tested using propensity score matching (PSM) and landmark analysis. A smooth curve technique was used to explore the shape of dose-response relationship.

**Results:**

Compared with the non-antibiotic group, antibiotic exposure was inversely associated with the mortality in the antibiotic group after adjustment for demographic and other potential confounders (a history of medication: HR, 0.650(95%CI: 0.360 to 1.173); an increase per day: HR, 0.967(CI: 0.924 to 1.011); 1–6 days: HR, 0.859(CI: 0.441 to 1.674); 7–40 days: HR, 0.474(CI: 0.225 to 0.999); P for trend = 0.040). A test for the interaction between sex was statistically significant (p = 0.016). A similar result was found as measured by landmark and PSM analysis. Significant negative dose-response relationship was shown by smooth curve analysis in the male patients but not female after adjustment for confounders(p = 0.028). No association was found between the antibiotic medication and adverse events of bevacizumab.

**Conclusion:**

Antibiotic exposure could be inversely associated with the mortality in mCRC patients treated with bevacizumab.

## Introduction

Colorectal cancer (CRC) is the third most commonly diagnosed cancer and the second leading cause of cancer-related death worldwide[1]. In advanced CRC patients, bevacizumab plus 5-fluorouracil-based or platinum-based therapy has become one of the standard first-line chemotherapy regimen for its significant clinical benefit[2–4]. Bevacizumab therapy results in adverse events including bleeding, hypertension, thrombosis and proteinuria[2,3]. Response to chemotherapy results from a complex interplay between gene regulation and environment. The microbiota is associated with CRC development[5] via an impact on intestinal inflammation[6] and chemoresistance to the treatment of CRC by modulating autophagy[7]. Evidence is accumulated that gut microbiota modulates the efficacy and toxicity of chemotherapy[8,9] and immunotherapy[10]. In a tumor-bearing mice model, mice that were germ-free or that had been treated with antibiotics (ATB) showed resistance to cyclophosphamide via modulating the anticancer immune response[9]. Similar results were observed in the cases of oxaliplatin[11] and irinotecan therapy[12]. In another preclinical study, tumors in antibiotic-treated or germ-free mice did not respond to CTLA blockade[10] and anti-PD-1 antibody [13]. Similar results were shown in the subsequent observational studies involving patients with non-small cell lung cancer or renal cell cancer[14,15].

On the contrary, some antibiotics, such as erythromycin, showed chemopreventive effects on mice with colorectal cancer[16]. However, in a Fusobacterium-positive mice model of colorectal cancer, oral metronidazole but not erythromycin significantly reduced Fusobacterium load and overall tumor growth[17]. A potential mechanism was that F. nucleatum modulated a molecular network of the Toll-like receptor, micro-RNAs, and autophagy to promote the colorectal cancer chemoresistance [7]. Interestingly, in the mouse model of age-related macular degeneration in its neovascular form, high-fat diet modulated gut microbiota and exacerbated choroidal neovascularisation through the overexpression of interleukin-6, interleukin-1b, tumor necrosis factor-a, and vascular endothelial growth factor A[18]. The above findings suggest that antibiotic medication may contribute variably to clinical outcomes in different cancers. Notably, it remains unclear whether antibiotic exposure affects the clinical outcomes in mCRC patients. In the current study, we retrospectively investigated the association between the antibiotic medication and the clinical outcomes in mCRC patients treated with bevacizumab.

## Patients and methods

mCRC patients treated with bevacizumab-containing chemotherapy at the Fuzhou General Hospital of Nanjing Military Command between January, 2009 and October, 2017 were enrolled. The follow-up visit was on February 28th, 2018. The exclusion criteria were: no pathological diagnosis, no measurable metastatic tumors, and missing drug prescription data. We collected data of demographics, medical history, drug prescriptions and disease outcomes. Because the data were anonymous, the requirement for informed consent was waived. This retrospective, single-center cohort study was approved by the Ethics Committee of Fuzhou General Hospital of Nanjing Military Region.

### Definition and measuring

Antibiotic exposure was defined as oral or intravenous medication of one of the seven antibiotic classes including penicillins, cephalosporins, macrolides, tetracyclines, sulphonamides, quinolones, and nitroimidazoles (excluding skin creams, mouthwash, topical antibiotic or antituberculosis). Antibiotics from drugstores outside the hospital were not taken into consideration. Cumulative duration of antibiotic medication in each chemotherapy period was calculated, which included the time of all antibiotic classes. Overall survival (OS) was defined as the time from the bevacizumab treatment to the death due to any cause or the last outpatient visit. Through telephone follow-up, the missing data were obtained as completed as possible.

To simplify the classification, left CRC was defined as rectal or sigmoid cancer and left transverse colon cancer while right CRC as right transverse colon, ascending colon and appendiceal cancers. In the subgroup of treatment line, the 1st+2nd line was defined as bevacizumab across 1st and 2nd lines of treatment beyond progression.

Hypertension was graded according to the common toxicity criteria for adverse events version 4.03 (CTCAE v4.03) as follows: Grade 1, prehypertension (systolic BP 120–139 mm Hg or diastolic BP 80–89 mmHg); Grade 2, stage 1 hypertension (systolic BP 140–159 mm Hg or diastolic BP 90–99 mmHg), medical intervention indicated, recurrent or persistent (> = 24 h), symptomatic increase by >20 mmHg (diastolic) or to >140/90 mmHg if previously within normal limits, monotherapy indicated; Grade 3, stage 2 hypertension (systolic BP > = 160 mm Hg or diastolic BP > = 100 mm Hg), medical intervention indicated, more than one drug or more intensive therapy than previously used indicated; Grade 4, life-threatening consequences (e.g., malignant hypertension, transient or permanent neurologic deficit, hypertensive crisis), urgent intervention indicated; Grade 5, death.

### Statistical analysis

The baseline characteristics of the study group and the control group were compared using the chi-square test for categorical variables and the Kruskal-Wallis test for continuous variables.

The Kaplan–Meier curves was used to estimate the association between antibiotic exposure and OS and the log-rank test was used for comparisons between two groups. The Cox proportional hazards models performed the univariate analysis with antibiotic exposure status and baseline characteristics. Variables that produced >10% change in the regression coefficient of antibiotic medication after they were introduced into the basic model and removed from the full model(S1 Table) were included in multivariate analysis. In the multivariate analysis, three models of Cox proportional hazard analyses were performed to determine the hazard for OS by the duration of antibiotic exposure:(1) never vs ever; (2) never vs 1–6 days and 7–40 days;(3) increase per day. The hazard ratio was adjusted for demographic characteristics (age, sex, BMI, ECOG score) and tumor parameters (No. of metastatic organs and differentiation degree) in sequence. The median duration of antibiotic exposure was 6 days (range, 1 to 40 days), so that 6 days was used as the cut-off value of subgroup (S1 Fig). We performed tests for linear trend by entering the median value of each class of antibiotic medication as a continuous variable in the models. Additionally, we used stratified analysis to examine the hazard ratio in the subgroup with age, sex and major interventional variables (surgery of primary site, line of treatment). The results were presented as hazard ratios (HR) with 95% confidence intervals (95%CI).

To further test the association between the mortality in mCRC and the antibiotic exposure, we conducted a sensitivity analysis using propensity score (PS) matching. Antibiotic medication status (no history and a history) were matched in a 1:1 ratio based on propensity scores (greedy-matching algorithm), with a calliper width equal to 0.02 of the standard deviation of the logit of the propensity score. The propensity score for antibiotic medication status was estimated by a Cox proportional hazard model, including demographic characteristics (age, sex, BMI, ECOG score) and tumor parameters (No. of metastatic organs, primary site and differentiation degree) as predictors. Further details on the PS analysis and matching methods were listed in S2 Table and S2 Fig in Supplemental data. Additionally, we performed another sensitivity analysis to assess the hazard ratio of death, excluding patients with OS less than three months, who were in the deleted subgroup 75% (22/29) lost to follow-up. This landmark method was also applied to avoid immortal time bias(15). Lastly, a smooth curve technique was used to study the shape of the relationship of antibiotic medication with the logarithm of the hazard risk (log HR) of mortality through a restricted cubic spline regression.

A 2-tailed P<0.05 was considered to be statistically significant in all analyses. Empower(R) (www.empowerstats.com; X&Y solutions, Inc., Boston MA) were used for all statistical analyses.

## Results

### Baseline characteristics and clinical outcomes

Between January, 2009 and October, 2017, 154 consecutive patients were screened in the electronic health record system, and 147 cases were included. Last date of data collection was February 28^th^, 2018, and by that time, 59 (40.1%) patients were reported of death. The median follow-up duration was 11.6 months (range, 0.2 to 52.9) for the deceased patients and 6.9 months (range, 0.1 to 57.8) for those who were censored. Reasons for the exclusion of 7(4.5%) patients were missing drug prescriptions data. The comparison of baseline characteristics between the included and the excluded was shown in S3 Table. Patient baseline characteristics were shown in Table 1.

**Table 1:** Baseline characteristics of patients with/without antibiotic exposure.

	Antibiotic Exposure		
	No(n = 86)	Yes(n = 61)	P-value
Mean age (SD), y	53.7 ± 12.3	58.6 ± 12.1	0.018
Mean BMI (SD), kg/m2	22.1 ± 3.8	22.3 ± 4.6	0.800
Sex,%			0.851
Female	38 (44.2%)	26 (42.6%)	
Male	48 (55.8%)	35 (57.4%)	
WHO performance status,%			0.377
0–1	43 (50.0%)	26 (42.6%)	
2–4	43 (50.0%)	35 (57.4%)	
Differentiation,%			0.400
No/Low	18 (20.9%)	10 (16.4%)	
Middle/High Not Record	53 (61.6%) 15 (17.4%)	44 (72.1%) 7 (11.5%)	
Primary Site,%			0.822
Right	24 (27.9%)	16 (26.2%)	
Left	62 (72.1%)	45 (73.8%)	
Surgery of primary organs,%			0.936
None	22 (25.6%)	17 (27.9%)	
Palliative	20 (23.3%)	13 (21.3%)	
Radical	44 (51.2%)	31 (50.8%)	
No. of metastatic sites,%			0.251
1	39 (45.3%)	20 (32.8%)	
2	25 (29.1%)	19 (31.1%)	
≥3	22 (25.6%)	22 (36.1%)	
Line of treatment,%			0.015
1st	45 (52.3%)	26 (42.6%)	
2nd	13 (15.1%)	8 (13.1%)	
1st+2nd	7(8.1%)	17 (27.9%)	
3rd-5th	21 (24.4%)	10 (16.4%)	
Chemotherapy used %			0.575
capeOX/FOLFOX	43 (50.0%)	32 (52.5%)	
FOLFIRI	20 (23.3%)	10 (16.4%)	
Others	23 (26.7%)	19 (31.1%)	

Data are Mean+SD / N(%). BMI = body mass index. capeOX = capecitabine+oxaliplatin. FOLFOX = oxaliplatin+fluorouracil+calcium folinate. FOLFIRI = Irinotecan+fluorouracil+calcium folinate. Differences in antibiotic exposure for the variables in the table were compared using the chi-square test for categorical measures and Kruskal-Wallis Test for continuous measures.

Data of antibiotic exposure and response were available in 147 of these patients. The median duration of chemotherapy was 8.3 months (range, 0.1 to 57.8 months). For patients who received first-line bevacizumab-containing treatment (n = 71), the median duration of therapy was 6.5 months (range, 0.1 to 57.8). It was 8.3 (range, 1.4 to 52.9) and 5.3(range, 0.1 to 26.8) months for those who received second-line (n = 21) and beyond (n = 31), respectively. 24 patients who received 1st+2nd line therapy had a median overall survival of 20.4 months. 75 patients (51.0%) received bevacizumab with FOLFOX or capeOX, and the rest of the patients received bevacizumab combined with FOLFIR(n = 30), or other therapy (n = 42). Hypertension was the major bevacizumab-associated adverse events, and the difference in adverse events between the antibiotic group and the control was not significant statistically (Table 2).

**Table 2:** Adverse events of bevacizumab therapy between the groups with/without antibiotic exposure.

	Antibiotic Exposure		P-value
	No (n = 86)	Yes (n = 61)	
Hypertension (CTC)			0.723
normal	32 (37.2%)	20 (32.8%)	
CTC 1–2	43 (50.0%)	29 (47.5%)	
CTC 2–4	10 (11.6%)	11 (18.0%)	
Not Record	1 (1.2%)	1 (1.6%)	
Proteinuria			0.39
No	83 (96.5%)	57 (93.4%)	
Yes	3 (3.5%)	4 (6.6%)	
Intestinal perforation			0.171
No	86 (100.0%)	59 (96.7%)	
Yes	0 (0.0%)	2 (3.3%)	
Thrombosis			0.171
No	86 (100.0%)	59 (96.7%)	
Yes	0 (0.0%)	2 (3.3%)	
Bleeding			1.000
No	85 (98.8%)	60 (98.4%)	
Yes	1 (1.2%)	1 (1.6%)	

Differences in antibiotic exposure for the variables in the table were compared using Fisher's exact probability test.

A total of 61 patients were identified of antibiotic exposure, including the first-line chemotherapy group (n = 26), the second-line group (n = 8), the beyond second-line group (n = 9) and the first- plus second-line group(n = 17). For these patients, β-lactams±inhibitors and quinolones were the most commonly administered antibiotics to manage the 75 infectious events. Only three infectious events were life-threatening, which were septicemia, intestinal perforation and intestine obstruction concomitant with peritonitis. As shown in S3 Fig, no significant difference was observed in overall survival between each line of treatment between the antibiotic group and the control group.

### Association between the antibiotic exposure and the mortality

Univariate and multivariate analysis was performed to explore the association between the antibiotic exposure and the mortality. The results of univariate analysis were shown in S4 Fig. After adjusted for age, sex, BMI and ECOG score, the HR for death was 0.926 (95%CI: 0.491, 1.748) in individuals with antibiotic exposure < 7 days, 0.511(95%CI: 0.251, 1.040) in subgroup > = 7 days compared with those in the control and 0.699 (95%CI: 0.405, 1.208) in patients with ATB exposure (ever vs never). After progressive adjustment for primary site parameters (No. of metastatic organs and differentiation degree), ATB exposure > = 7 days was significantly associated with a lower HR for death (HR, 0.474; 95%CI: 0.225, 0.999). Moreover, in this model, we found a significant linear trend between the days of ATB use and the HR for death (p = 0.046 for trend). For a per day increase in antibiotic exposure, the HR for death was 0.967(95%CI: 0.924,1.011) in the final multivariable model adjusted for primary site parameters (Table 3).

**Table 3:** Antibiotic use and multivariate HR of overall survival with 95% CIs in mCRC.

	Antibiotic Exposure (vs. None)		*P* for trend	Antibiotic used (vs. never)	Increase per day
	1–6 d	7–40 d			
**Primary Cohort**					
Deaths/ Patients	16/32	13/29	*-*	29/61	59 /147
Not Adjusted	1.039 (0.570, 1.896)	0.671 (0.342, 1.319)	0.242	0.849 (0.507, 1.421)	0.981 (0.944, 1.019)
Model 1*	0.926 (0.491, 1.748)	0.511 (0.251, 1.040)	0.061	0.699 (0.405, 1.208)	0.970 (0.930, 1.012)
Model 2#	0.859 (0.441, 1.674)	0.474 (0.225, 0.999)	0.046	0.650 (0.360, 1.173)	0.967 (0.924, 1.011)
**Landmark Cohort**					
Deaths/ Patients	11/26	11/26	*-*	26/52	52/118
Not Adjusted	1.007 (0.530, 1.912)	0.668 (0.328, 1.359)	0.260	0.829 (0.480, 1.434)	0.983 (0.945, 1.023)
Model 1*	0.897 (0.451, 1.783)	0.498 (0.235, 1.056)	0.066	0.672 (0.374, 1.209)	0.973 (0.931, 1.016)
Model 2#	0.857 (0.418, 1.758)	0.483 (0.219, 1.065)	0.067	0.652 (0.346, 1.230)	0.972 (0.928, 1.018)
**PS-Matched Cohort**					
Deaths/ Patients	15/29	11/28	-	26/47	47/114
Not Adjusted	1.065 (0.544, 2.083)	0.667 (0.320, 1.388)	0.255	0.848 (0.475, 1.515)	0.981 (0.942, 1.022)

Landmark Cohort contains the initial populations whose overall survival more than 3 months. Data are Hazard risk (95% CI). BMI indicates body mass index; and mCRC, metastatic colorectal cancer.

*Adjusted for age(<60y, ≥60y), sex, BMI (trisection) ECOG score (0–1, 2–4).

#In addition to age(<60y, ≥60y),sex, BMI (trisection), ECOG score (0–1, 2–4),this model is further adjusted for No. of metastatic organs(1, 2, ≥3) and differentiation degree(no/low, middle/high, not record).

**Fig 1:**
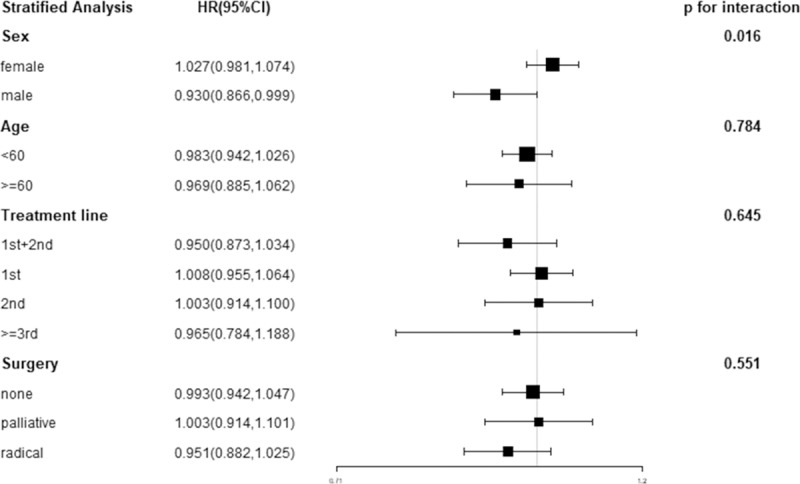
The stratified analysis for unadjusted HR in the subgroups of age, sex, surgery of primary site and line of treatment. HR(vs. continued) = hazard ratio.

When stratified by sex, the crude HR was 0.930(95%CI:0.866, 0.999) for the male and 1.027(CI: 0.981, 1.074) for the female, respectively. A test of HR for death based on the interaction between sex and duration of antibiotic medication was statistically significant (p = 0.016). HRs were inverse between the subgroup of surgery of primary site and line of treatment but not significantly, which was shown in Fig 1.

### Sensitivity analyses

To further confirm the association between antibiotic medication and HR for death, PS-matched cohort and landmark cohort were performed as the sensitivity analysis. In the sensitivity analysis among the PS-matched population, HR for death (vs. no ATB) was 1.065(95%CI: 0.544 to 2.083) for patients with a exposure history of 1 to 6 days and 0.667 (CI: 0.320, 1.388) for those with a exposure history of 7–40 days. Antibiotic exposure was inversely and significantly associated with the risk for death in these patients (p for trend = 0.255). The C-statistic for the model was 0.654.

In the analyses for landmark cohort excluding the patients with an overall survival less than 3 months, HR for death (vs. no ATB) was 0.829(95%CI: 0.480 to 1.434) for not adjusted model, 0.672(CI:0.374, 1.209) for crude model and 0.652(CI:0.346, 1.230)for adjusted model. However, a linear trend between the days of ATB use and the HR for death was not significant (p = 0.067 for trend) in the adjusted model. These results were consistent with that in the initial analyses (Table 3).

**Fig 2:**
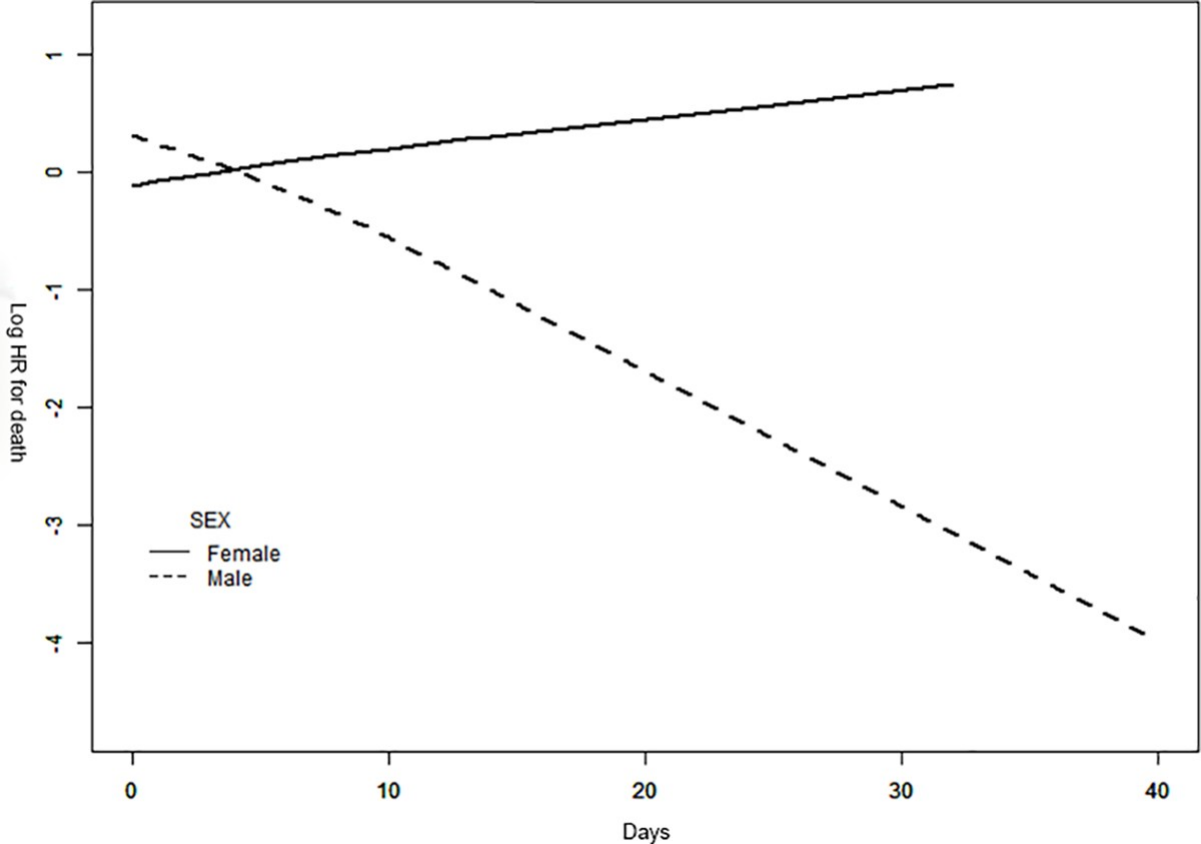
Smooth curves between antibiotic use and time to mortality stratified by sex. The model adjusted for age(<60y, ≥60y), BMI (trisection) ECOG score (0–1, 2–4). P = 0.028 for likelihood ratio test in the male group. HR indicates hazard risk.

Finally, in the smooth curve analysis stratified by sex, the curves were separated markedly when the days of the antibiotic use was growing. After adjusted for age, ECOG score and BMI, a significant negative dose-response relation was observed in the male group (P = 0.028) (Fig 2).

## Discussion

In this retrospective, hospital-based cohort study, we found an inverse association between the antibiotic exposure and the mortality in male mCRC patients treated with bevacizumab therapy. A significant linear trend was observed between days of antibiotic use and hazard risk for death when adjusted for the potential confounders. There was no association between the antibiotic exposure and the adverse events of bevacizumab therapy.

To the best of our knowledge, this is the first retrospective study to preliminarily verify the association between the antibiotic exposure and the clinical outcomes in mCRC patients. Overall, our finding of a decreased risk for death induced by antibiotic use is consistent with preclinical studies[7,17] and suggests that the antimicrobial intervention is a feasible treatment for patients with metastatic colorectal cancer. A similar result were also shown in pancreatic cancer, another malignant carcinoma of the digestive system. In a preclinical study, broad-spectrum oral antibiotic protected against preinvasive and invasive pancreatic ductal adenocarcinoma(PDA) and enabled efficacy for immunotherapy by upregulating PD-1 expression in a PDA-bearing mouse model[19].

Interestingly, in another mouse model with K-ras mutation and p53 loss, we also observed that depletion of microbiota significantly suppressed lung tumor growth[20]. However, clinical evidence to the contrary has been observed that patients with cancer and sepsis had a higher hospital mortality than the cancer-free group. A possible explanation is that our definition of antibiotic exposure has eliminated most life-threatening infective events.

Previous studies showed numerous sex-based differences in the clinical outcomes of colorectal cancer patients, including morbidity and overall survival and estrogen might be a potential protective factor[21]. However, there were few studies on sex-based differences in the clinical outcomes of colorectal cancer patients with antibiotic medication. In our study, the sex-based difference was found in the hazard ratio for death. With the increase in days of antibiotic use, a significant negative dose-repose relationship was found in male patients but not female ones. Additionally, microbiota may play a vital role in the mechanism for sex-based difference in the mortality in mCRC patients.

The current study had long follow-up period of eight years and was a real-world observation to assess the drug-influenced mortality in mCRC. To reduce the bias in this retrospective observational study, we used multivariate cox proportional hazard models with statistical adjustment for confounders. Furthermore, the impact residual confounders were minimized by PS-matching analysis. We performed a landmark analysis to control the immortal time bias. Finally, a smooth curve technique was used to explore the shape of dose-response relation.

The study had limitations. It was a small-scale, single-center study. Therefore, bias could be caused by residual and unmeasured confounders. Despite the loose inclusion criteria and the long-term follow-up, there were only 154 mCRC patients treated with bevacizumab, and 147 patients are included finally. Moreover, immortal time and attrition bias were unavoidable. However, a landmark cohort analysis showed that the antibiotic-mortality association was robust in all models. Future studies are needed to examine associations based on specific classes of antibiotic and simplified chemotherapy regimens in randomized control trials.

## Conclusions

In summary, longer duration of antibiotic exposure could be associated with a lower risk of death in mCRC patients treated with bevacizumab. Moreover, there was a significant negative dose-repose relation in male patients but not female after adjustment for confounders.

## Supporting information

S1 Fig Distribution of antibiotic use(increased per day) in overall and male/female group. Dotted line indicates the median of antibiotic exposure time at 6 days.
(TIF)

S2 Fig Distribution of propensity-matching antibiotic use status (yes vs no) groups after matching. (PNG)

S3 Fig Overall survival and each line of treatment in metastatic colorectal cancer. (TIF)

S4 Fig Univariate analysis for risk factors of overall survival in metastatic colorectal cancer. BMI was divided into trisection, that is low, moderate and high group; ATB = Antibiotic; BMI = Body Mass Index.
(TIF)

S1 Table The regression coefficient between antibiotic us and the death(increase per day). The value of β is adjusted for age and sex.
(DOCX)

S2 Table After propensity-matching antibiotic use status (no vs. yes) groups. Data are Mean+SD / N(%); BMI = body mass index; PS = propensity score; For categorical variables: N(%); Standardized difference = abs(P1-P0)/sqrt((P1*(1-P1)+P0*(1-P0))/2); Differences in antibiotic exposure for the variables in the table were compared using the chi-square test.
(DOCX)

S3 Table Baseline characteristics of patients with/without complete prescription record. Data are Mean+SD / N(%); BMI = body mass index; capeOX = capecitabine +oxaliplatin; FOLFOX = oxaliplatin+fluorouracil+calcium folinate; FOLFIRI = Irinotecan+fluorouracil+calcium folinate; Differences in prescription record status were based on the chi-square test for categorical measures and Kruskal-Wallis Test for continuous measures.
(DOCX)
