# Supplementary figures and images for: Association of antibiotic exposure with the mortality in metastatic colorectal cancer patients treated with bevacizumab-containing chemotherapy: A hospital-based retrospective cohort study
Source: PLoS One. 2019 Sep 10;14(9):e0221964. doi: 10.1371/journal.pone.0221964 (PMC6736303; doi:10.1371/journal.pone.0221964)

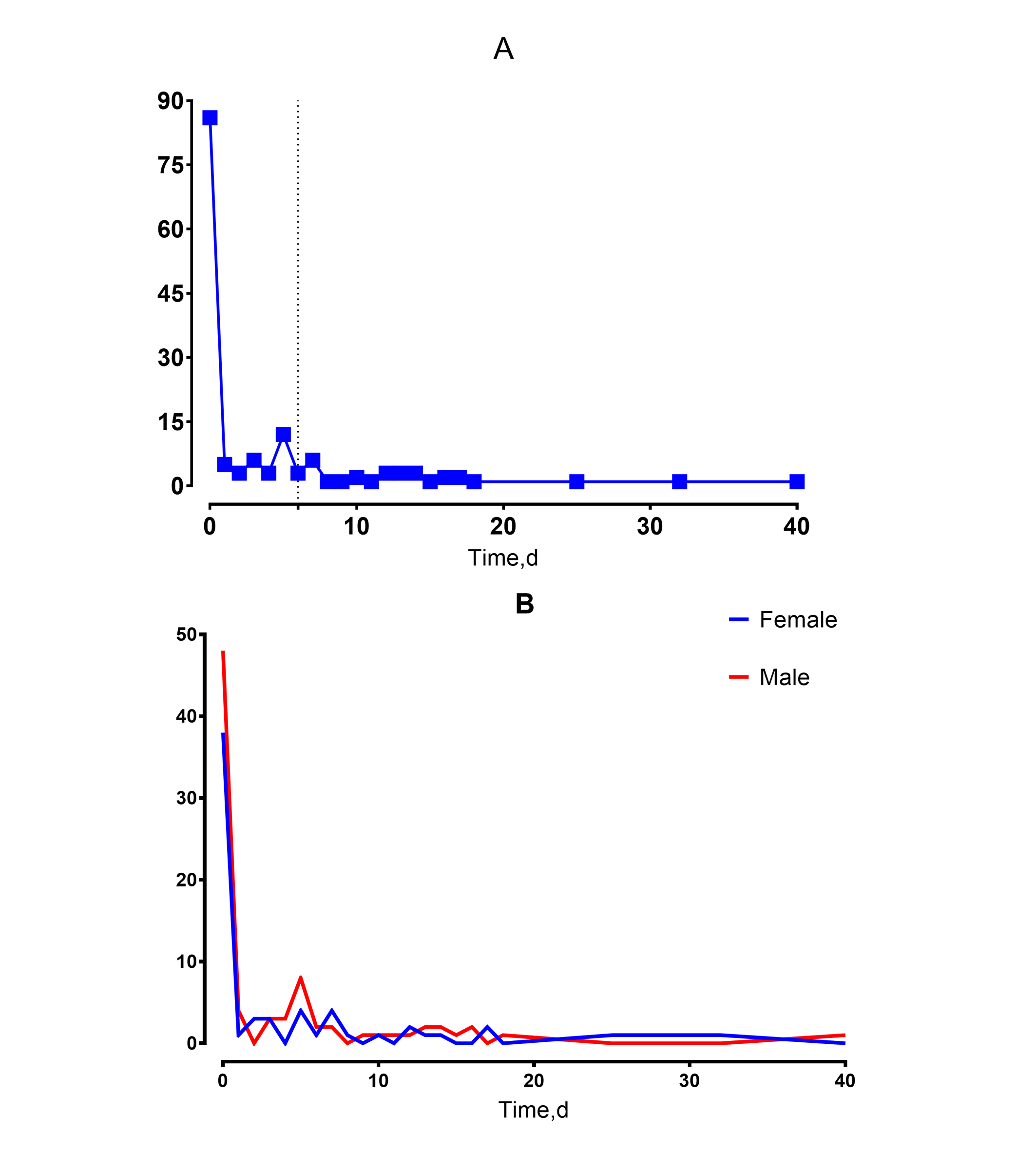

Supplement: S1 Fig — Dotted line indicates the median of antibiotic exposure time at 6 days. (TIF) [file pone.0221964.s001.tif]

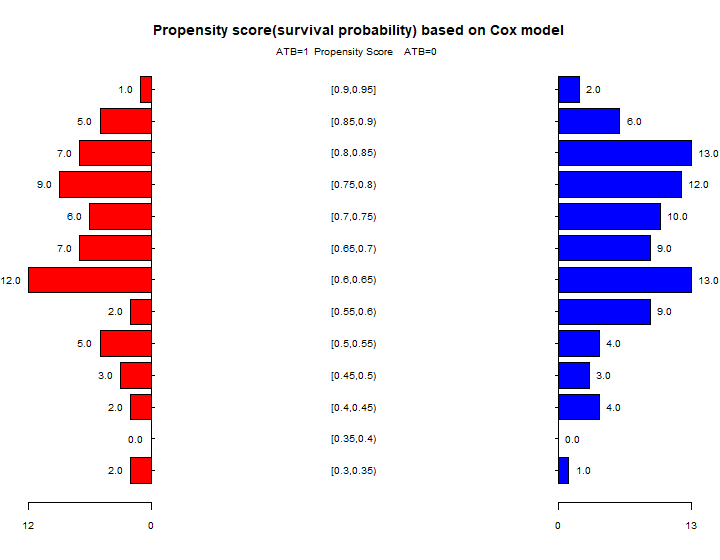

Supplement: S2 Fig — (PNG) [file pone.0221964.s002.png]

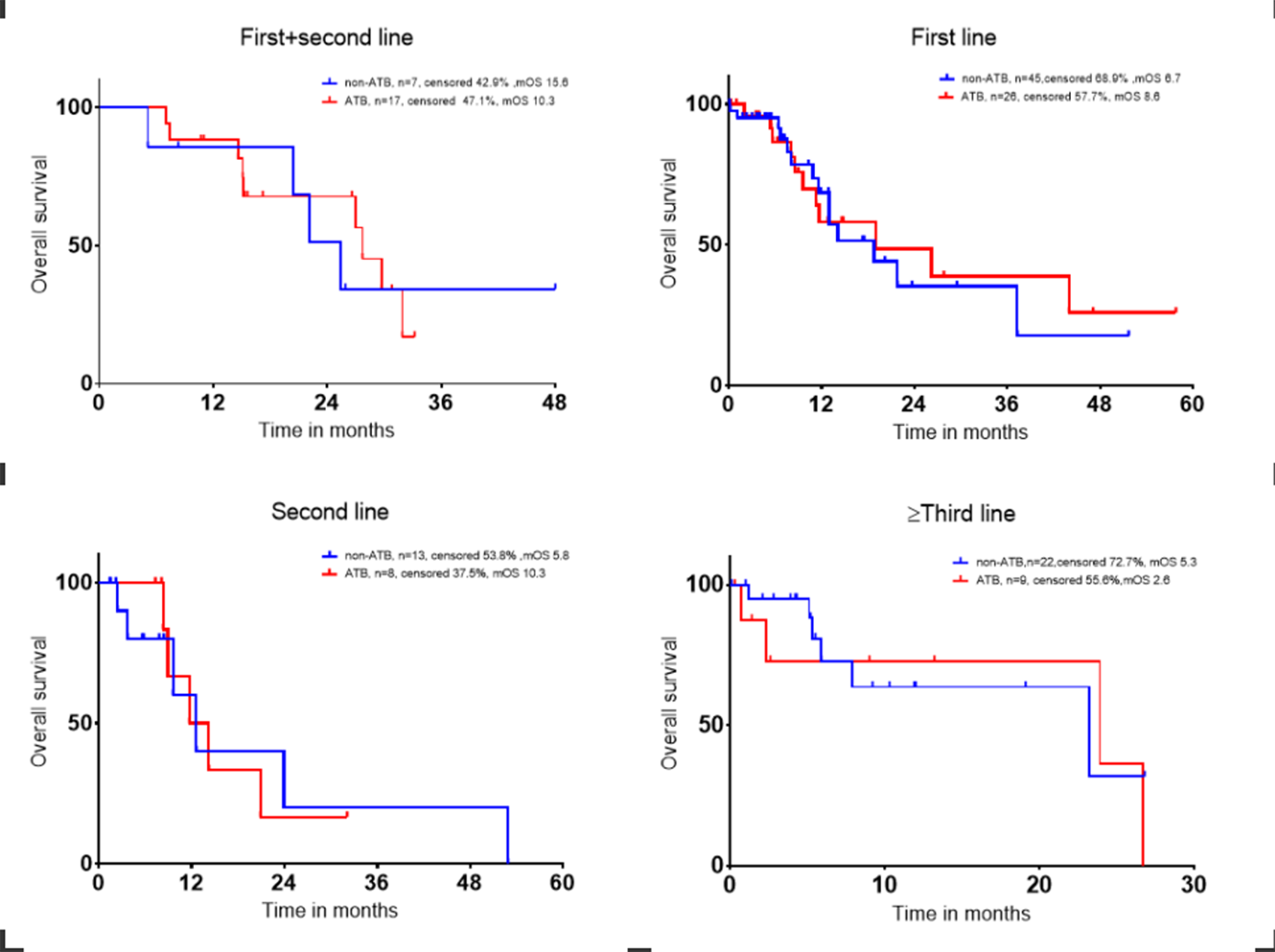

Supplement: S3 Fig — (TIF) [file pone.0221964.s003.tif]
