# Supplementary material for: Association of antibiotic exposure with the mortality in metastatic colorectal cancer patients treated with bevacizumab-containing chemotherapy: A hospital-based retrospective cohort study
Source: PLoS One. 2019 Sep 10;14(9):e0221964. doi: 10.1371/journal.pone.0221964 (PMC6736303; doi:10.1371/journal.pone.0221964)
Supplement: S1 Table — The value of β is adjusted for age and sex. (DOCX) [file pone.0221964.s005.docx]

|  | Basic model | Δβ% | Full model | Δβ% |
| --- | --- | --- | --- | --- |
| BMI | -0.0289 | 42.4 | -0.0232 | 15.6 |
| ECOG | -0.0219 | 7.9 | -0.0308 | 12.0 |
| Site | -0.0214 | 5.4 | -0.0246 | 10.5 |
| Differentiation | -0.0179 | 11.8 | -0.0255 | 7.3 |
| Metastatic | -0.0275 | 35.5 | -0.0209 | 24.0 |
| Line | -0.0146 | 28.1 | -0.038 | 38.2 |
| Surgery | -0.0226 | 11.3 | -0.0307 | 11.6 |
