# Supplementary material for: Association of antibiotic exposure with the mortality in metastatic colorectal cancer patients treated with bevacizumab-containing chemotherapy: A hospital-based retrospective cohort study
Source: PLoS One. 2019 Sep 10;14(9):e0221964. doi: 10.1371/journal.pone.0221964 (PMC6736303; doi:10.1371/journal.pone.0221964)
Supplement: S2 Table — Data are Mean+SD / N(%); BMI = body mass index; PS = propensity score; For categorical variables: N(%); Standardized difference = abs(P1-P0)/sqrt((P1*(1-P1)+P0*(1-P0))/2); Differences in antibiotic exposure for the variables in the table were compared using the chi-square test. (DOCX) [file pone.0221964.s006.docx]

|  | Antibiotic use | | Standardized  difference | P value |
| --- | --- | --- | --- | --- |
|  | No(n=57) | Yes(n=57) |  |  |
| Sex,% |  |  | 0.1412 | 0.5727 |
| Female | 28 (49.1) | 24 (42.1) |  |  |
| Male | 29 (50.9) | 33 (57.9) |  |  |
| Age,% |  |  | 0.0728 | 0.8460 |
| <60 | 35 (61.4) | 37 (64.9) |  |  |
| >=60 | 22 (38.6) | 20 (35.1) |  |  |
| BMI ( trisection ),% |  |  |  | 0.5259 |
| Low | 18 (31.6) | 18 (31.6) | 0.0000 |  |
| Middle | 18 (31.6) | 23 (40.4) | 0.1836 |  |
| High | 21 (36.8) | 16 (28.1) | 0.1882 |  |
| WHO performance status,% |  |  | 0.0705 | 0.8508 |
| 0-1 | 27 (47.4) | 25 (43.9) |  |  |
| 2-4 | 30 (52.6) | 32 (56.1) |  |  |
| No. of metastatic sites,% |  |  |  | 0.5850 |
| 1 | 23 (40.4) | 20 (35.1) | 0.1087 |  |
| 2 | 14 (24.6) | 19 (33.3) | 0.1943 |  |
| ≥3 | 20 (35.1) | 18 (31.6) | 0.0745 |  |
| Differentiation,% |  |  |  | 0.1219 |
| No/low | 17 (29.8) | 8 (14) | 0.3887 |  |
| Middle/high | 35 (61.4) | 42 (73.7) | 0.2646 |  |
| Not Record | 5 (8.8) | 7 (12.3) | 0.1145 |  |
| Primary Site,% |  |  | 0.0387 | 1.0000 |
| Right | 40 (70.2) | 41 (71.9) |  |  |
| Left | 17 (29.8) | 16 (28.1) |  |  |
| Line of treatment,% |  |  |  | 0.0517 |
| 1st | 4 (7) | 15 (26.3) | 0.5361 |  |
| 2nd | 30 (52.6) | 25 (43.9) | 0.1762 |  |
| 1st+2nd | 9 (15.8) | 7 (12.3) | 0.1011 |  |
| 3rd-5th | 14 (24.6) | 10 (17.5) | 0.1728 |  |
| Surgery of primary sites,% |  |  |  | 0.5369 |
| None | 13 (22.8) | 16 (28.1) | 0.1211 |  |
| Palliative | 17 (29.8) | 12 (21.1) | 0.2024 |  |
| Radical | 27 (47.4) | 29 (50.9) | 0.0702 |  |
