# Supplementary material for: Association of antibiotic exposure with the mortality in metastatic colorectal cancer patients treated with bevacizumab-containing chemotherapy: A hospital-based retrospective cohort study
Source: PLoS One. 2019 Sep 10;14(9):e0221964. doi: 10.1371/journal.pone.0221964 (PMC6736303; doi:10.1371/journal.pone.0221964)
Supplement: S3 Table — Data are Mean+SD / N(%); BMI = body mass index; capeOX = capecitabine +oxaliplatin; FOLFOX = oxaliplatin+fluorouracil+calcium folinate; FOLFIRI = Irinotecan+fluorouracil+calcium folinate; Differences in prescription record status were based on the chi-square test for categorical measures and Kruskal-Wallis Test for continuous measures. (DOCX) [file pone.0221964.s007.docx]

|  | Complete Record | |  | |  | |  |
| --- | --- | --- | --- | --- | --- | --- | --- |
|  | NO | Yes | | P-value | |  | |
| Case,n | 7 | 147 | |  | |  | |
| Mean age (SD), y | 56.6 ± 9.3 | 55.7 ± 12.4 | | 0.855 | |  | |
| Mean BMI (SD), kg/m2 | 20.9 ± 1.1 | 22.2 ± 4.1 | | 0.419 | |  | |
| Sex,% |  |  | | 0.479 | |  | |
| female | 4 (57.1%) | 64 (43.5%) | |  | |  | |
| male | 3 (42.9%) | 83 (56.5%) | |  | |  | |
| WHO performance status,% |  |  | | 0.09 | |  | |
| 0-1 | 1 (14.3%) | 69 (46.9%) | |  | |  | |
| 2-4 | 6 (85.7%) | 78 (53.1%) | |  | |  | |
| Differentiation,% |  |  | | 0.437 | |  | |
| no/low | 2 (28.6%) | 28 (19.0%) | |  | |  | |
| middle/high | 3 (42.9%) | 97 (66.0%) | |  | |  | |
| Not Record | 2 (28.6%) | 22 (15.0%) | |  | |  | |
| Primary Site,% |  |  | | 0.937 | |  | |
| Right | 2 (28.6%) | 40 (27.2%) | |  | |  | |
| Left | 5 (71.4%) | 107 (72.8%) | |  | |  | |
| Surgery of primary sites,% |  |  | | 0.878 | |  | |
| None | 2 (28.6%) | 39 (26.5%) | |  | |  | |
| Palliative | 1 (14.3%) | 33 (22.4%) | |  | |  | |
| Radical | 4 (57.1%) | 75 (51.0%) | |  | |  | |
| No. of metastatic sites,% |  |  | | 0.159 | |  | |
| 1 | 5 (71.4%) | 59 (40.1%) | |  | |  | |
| 2 | 2 (28.6%) | 44 (29.9%) | |  | |  | |
| ≥3 | 0 ( 0.0%) | 44 (29.9%) | |  | |  | |
| Line of treatment,% |  |  | | 0.067 | |  | |
| 1st | 0 ( 0.0%) | 24 ( 16.3%) | |  | |  | |
| 2nd | 7 (100.0%) | 71 ( 48.3%) | |  | |  | |
| 1st+2nd | 0 ( 0.0%) | 21 ( 14.3%) | |  | |  | |
| 3rd-5th |  |  | |  | |  | |
| Chemotherapy used,% |  |  | | 0.342 | |  | |
| capeOX/FOLFOX | 3 (42.9%) | 75 (51.0%) | |  | |  | |
| FOLFIRI | 3 (42.9%) | 30 (20.4%) | |  | |  | |
| others | 1 (14.3%) | 42 (28.6%) | |  | |  | |
